# Bryophytes Floristic Patterns in the Sicilian Aquatic and Humid Habitats—Important Refuges for Biodiversity Safeguarding in the Mediterranean Islands

**DOI:** 10.3390/plants14020199

**Published:** 2025-01-12

**Authors:** Patrizia Campisi, Mattia Letizia Marino

**Affiliations:** 1 Department of Biological, Chemical and Pharmaceutical Sciences and Technologies, University of Palermo, via Archirafi 38, I 90123 Palermo, Italy; 2 NBFC–National Biodiversity Future Center, Piazza Marina 61 (c/o Palazzo Steri), 90133 Palermo, Italy; 3 Department of Agricultural, Food and Forest Sciences, University of Palermo, Viale delle Scienze, Bldg. 5, 90128 Palermo, Italy; mattialetizia.marino@unipa.it

**Keywords:** aquatic and humid areas, ecological traits, biodiversity loss, Mediterranean area

## Abstract

In this study, a focus on the populations of bryophytes living in aquatic and humid habitats of Sicily is presented. This investigation aims to evaluate the consistency and diversity of this group of taxa. The complete list of taxa known to date in these habitats is provided, with reference to hornworts, liverworts, and mosses, and the patterns related to the biological, ecological, and chorological features of this bryophyte flora are also illustrated. Since climate change is currently the major threat of extinction for many of these species growing in aquatic and humid environments, these taxa constitute a component of the flora worthy of particular interest in studies on the quantification of the impact of global warming on biodiversity. Overall, the analysis found that, with a total of 224 taxa, this group constitutes 36% of the Sicilian bryophyte flora; it is quite diversified from a taxonomic point of view, being represented by 55 families and 106 genera. Related to ecological features and reproductive biology, some aspects of possible vulnerability are highlighted, as well as others of resilience. Moreover, the importance of future monitoring of these taxa is underlined, also in consideration of the fact that some of them, such as *Sciuro-hypnum ornellanum*, are species of conservation interest in Italy and in Europe, and that four other taxa are threatened in Italy.

## 1. Introduction

As is known, in many areas of the planet, humid and aquatic environments have long been among the most impacted ecosystems, threatened by multiple anthropogenic factors which have in many cases determined their alteration, reduction, or even disappearance. As a consequence, they are protected by numerous directives at the international level, such as the Water Framework Directive (WFD 2000/60/EC) and the Ramsar Convention (Ramsar Convention Secretariat 2015). Given the high risk of rarefaction that these habitats face on a global scale, a risk that has recently increased due to the effects of climate change, increasing attention at a political, economic, and scientific level is being paid to them and to the species they host.

Bryophytes are undoubtedly among the organisms that characterize the biodiversity of these environments. As highlighted by Vitt and Glime [1], some species, such as *Fontinalis antipyretica* Hedw., can be considered obligate aquatic, while others are less dependent on the constant presence of water and are therefore considered facultative aquatic species.

The bryophyte species can generally live in all types of humid and aquatic habitats, such as rivers, lakes, pools, waterfalls, ponds, occupying different ecological niches, and depending on the greater or lesser ecological need for water, they can be distinguished into rheophilous, limnophilous, and semi-aquatic emergent species.

In general, these plants are strongly influenced by climatic and soil conditions and are very quickly affected by habitat changes. As a result, they have long been recognized as indicators of environmental quality and used in biomonitoring studies [2].

On the other hand, alongside the growing interest in this group of aquatic macrophytes, knowledge about bryophytes would require an implementation of more precise data on both species distribution and taxa ecological requirements, and on floristic composition as well in many geographical areas.

With reference to Europe, as recently highlighted by Rimac et al. [3], knowledge on the diversity, distribution, and ecological features of taxa is still heterogeneous or related to very limited single sites, with some countries, such as Bulgaria, Croatia, or north-western Portugal, better known than others. In the Mediterranean area, for example, some studies have taken into consideration the riparian bryophyte communities of some river environments in Portugal, Spain, Italy, France, Greece, Slovenia, and Cyprus [4]. A very high floristic and vegetation diversity emerged from such investigations, which highlights the territorial uniqueness of the different bryophyte communities, strongly associated with major climatic and water chemistry variables. Similar results also emerge from two other interesting surveys conducted in Croatia by Rimac et al. [3,5]. The authors provide a detailed picture of the bryophyte flora and communities of the running and standing waters of Dinaric and Pannonian ecoregions in this area, confirming the high peculiarity of the bryophyte component occurring in these habitats, strongly linked to the specific meso- and microclimatic conditions, and whose populations are greatly subjected to fragmentation.

Also, in Italy, the knowledge on bryophytes of humid and aquatic environments is still incomplete and fragmentary, as the information refers to a still minimal part of the Italian territory, often “hidden” in floristic lists related to large areas and not to specific aquatic or humid habitats. Among more recent works, it is worth mentioning a study on the mountain marshes of Tuscany [6] and research on the lake habitats and peat bogs of the Maritime Alps [7]. Later investigations have been carried out on the priority natural habitat (3170*) under the Habitats Directive 92/43/EEC (European Commission, 1992), exploring the Mediterranean temporary ponds in central and insular Italy [8,9,10,11,12] and the Marmore Waterfalls Regional Park in Umbria [13]. These studies are often conducted with the aim of also monitoring human impact and conserving sites at risk, and have often provided new information on the bryophyte flora of aquatic and humid habitats, on the distribution and ecology of the related taxa, and on the dynamics of the vegetation, underlining the opportunity to extend the research to the many areas of Italy not yet investigated.

As regards Sicily, a useful summary on the state of knowledge on the bryophytes of the watercourses of the island has been issued by Dia et al. [14]. This contribution provides a full picture of the available information about the bryophyte diversity of this habitat, based on an analysis of the literature data relating to over a century of investigations conducted in various river areas of the island. As highlighted in this contribution, the investigation and therefore the knowledge have been mostly directed towards the main watercourses of the north-eastern part of the island. More recently, some studies on the bryophytes of Sicilian humid environments have been started, and Campisi et al. [15] have illustrated the first results with reference to some temporary and permanent wetlands. Several taxonomic, chorological, biological, and ecological traits of these sites are highlighted, as well as the presence of some rare taxa in Sicily, such as those of the moss *Physcomitrium patens* (Hedw.) Mitt. The interest in these environments, which are too often at risk, and in the bryophyte component has also led to investigations conducted with an approach aimed at the animal and plant components, with protectionist purposes, as made known by Troia et al. [16]. This research has in many cases highlighted the presence of interesting taxa or flora, leading us once again to think that the knowledge on the bryophyte flora of aquatic and humid habitats also needs to be implemented for Sicily.

Taking this into account, recently, within the framework of the National Recovery and Resilience Plan (NRRP) funded by the EU at a national level and aimed at the census and monitoring of Italian biodiversity, some investigations have been started on the bryophyte species of Sicilian aquatic and humid environments, the first results of which are the subject of this study. These surveys aim to estimate the taxonomic consistency of the bryophyte flora of these environments, which is exposed, as mentioned, to a high risk in Sicily and in the Mediterranean area; give a view of its distribution on the island from a geographical aspect and also with reference to habitats; provide a view of its ecological features; acquire data on its reproductive potential; and identify the taxa of major conservation interest with reference to both Italy and Europe.

In this paper, an overview of the hygrophilous and hydrophilous bryophyte flora of Sicily is provided for the first time. Furthermore, the floristic patterns and the taxonomic, ecological, and chorological characteristics of such bryophyte flora are illustrated, as well as the taxa of conservation interest in the related interesting and fragile environments.

## 2. Results

**Figure 1:**
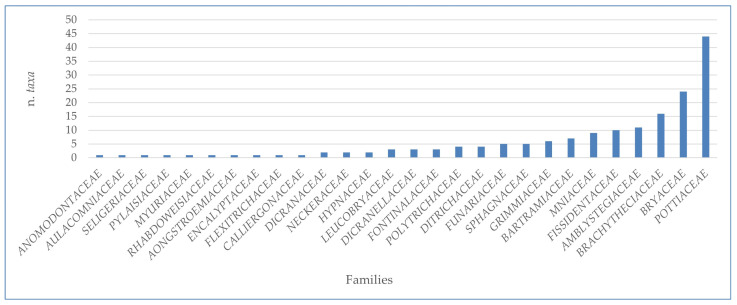
Taxonomic consistency of different families of mosses in the Sicilian aquatic and humid habitats.

**Figure 2:**
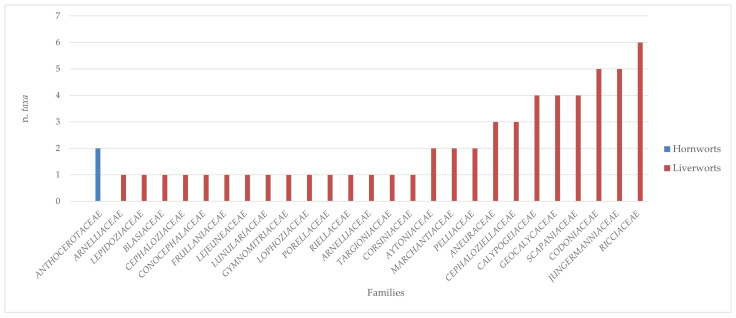
Taxonomic consistency of different families of hornworts and liverworts in the Sicilian aquatic and humid habitats.

**Figure 3:**
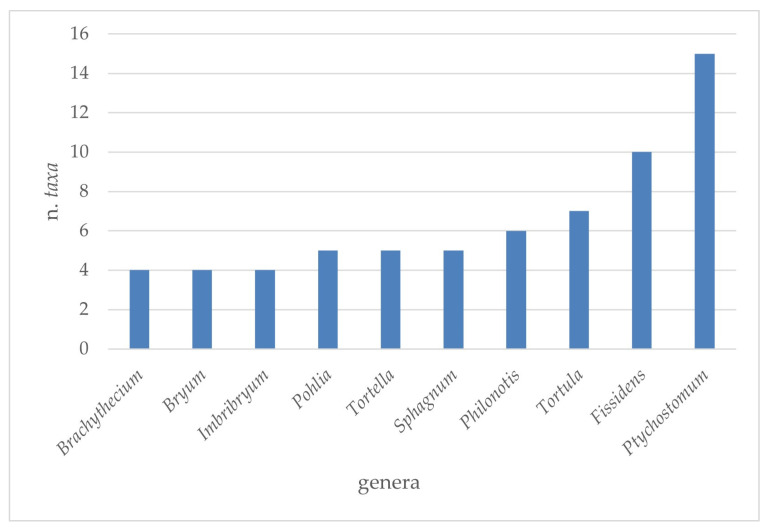
Taxonomic consistency of 10 more representative genera (with 4 or more taxa) of mosses of the Sicilian aquatic and humid habitats.

**Figure 4:**
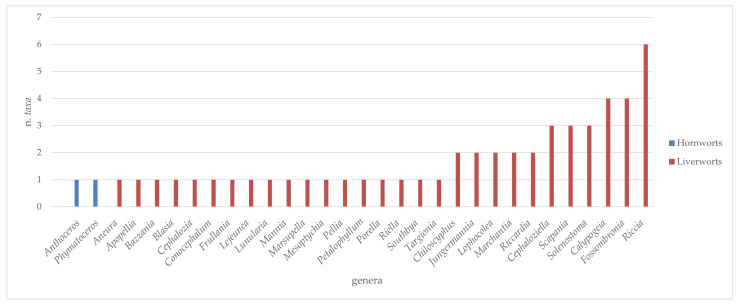
Taxonomic consistency of different genera of hornworts and liverworts of Sicilian aquatic and humid habitats.

The analysis conducted on the species of Sicilian aquatic and humid habitats allowed us to extrapolate a complex of 224 taxa (v. Supplementary File S1), of which 167 were mosses, 55 liverworts, and 2 hornworts (Table 1). Overall, these taxa belong to 28 families and 75 genera of mosses; 26 families with 30 genera of liverworts; and 1 family with 2 genera of hornworts (Table 1 and Figure 1, Figure 2, Figure 3 and Figure 4). There are many families that were represented only by a few taxa (from 1 to 3), while greater numbers of species were found for the *Pottiaceae*, *Bryaceae,* and *Brachytheciaceae* families (43, 21, and 16 taxa, respectively) for the mosses (Figure 1), and for *Ricciaceae* with 6 taxa, and *Codoniaceae* and *Jungermanniaceae*, both represented by 5 taxa, for the liverworts (Figure 2). Within the large number of moss genera, the majority (38 genera) were represented by only one taxon; many genera were represented by 2 or 3 taxa (15 and 11, respectively), and 10 genera contributed to the flora with 4 taxa or more. These genera are divided into the following seven families: *Bartramiaceae*, with *Philonotis*; *Brachytheciaceae* with *Brachythecium*; *Bryaceae*, with *Bryum*, *Imbribryum,* and *Ptychostomum*; *Fissidentaceae*, with *Fissidens*; *Mniaceae*, with *Pohlia*; *Pottiaceae*, with *Tortella* and *Tortula*; and *Sphagnaceae* with *Sphagnum* (Figure 3). For liverworts and hornworts, the genus *Riccia*, with six taxa, and the genera *Calypogeia* and *Fossombronia*, both presented with four species, stand out for their species richness (Figure 4).

**Figure 5:**
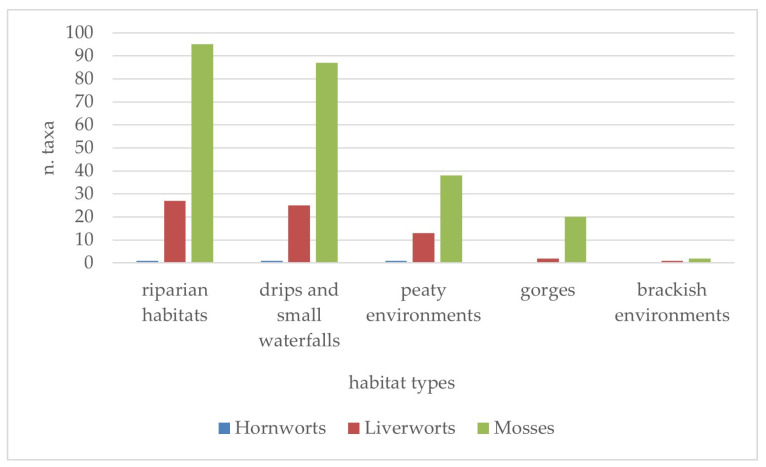
Presence of Sicilian bryophyte taxa in different aquatic and humid habitat types.

The survey highlights that the bryophytes identified so far in Sicilian aquatic environments and wetlands live primarily in three types of habitats (Figure 5): riparian environments of watercourses and lakes, where a total of 123 taxa were found (1 hornwort, 27 liverworts, and 95 mosses); drips and small waterfalls, where 113 species were represented (1 hornwort, 25 liverworts, and 87 mosses); and peat environments, represented mostly by the small peat bog remnants of the Madonie mountain range, which appear to host 52 taxa (1 hornwort, 13 liverworts, and 38 mosses). Finally, gorges and brackish water environments contributed to the overall bryofloristic richness, with 22 (2 liverworts and 20 mosses) and 3 (1 liverwort and 2 mosses) taxa, respectively. In all these habitats, the presence of both mosses and liverworts was recorded with differences in species richness also correlated to the overall consistency of the three groups of bryophytes.

The analysis of the distribution of the *taxa* under study on the island also allowed us to highlight five taxa that are particularly interesting as they are very rare and at risk of extinction in Italy [17,18] or even in Europe [19]. They are the following: *Sciuro-hypnum ornellanum* (Molendo) Ignatov & Huttunen, assessed as Critically Endangered (Possibly extinct) CR(PE); *Riella notarisii* (Mont.) Mont., and *Weissia levieri* (Limpr.) Kindb., considered Endangered (EN); and *Hydrogonium bolleanum* (Müll.Hal.) A.Jaeger and *Tortula freibergii* Dixon & Loeske, both assessed as Vulnerable (VU).

*Sciuro-hypnum ornellanum* has a very fragmented distribution in Italy, being reported only from Veneto, in the northern part of the peninsula, and from the Peloritani Mountains in Sicily. It has not been recorded for over 50 years and further investigations, despite having been conducted, have not provided new information on the only known subpopulations. Therefore, we think that there is a high probability that it may already be extinct in Italy. Furthermore, it is a taxon considered at risk at a European level also, qualified for the EN category [19].

The liverwort species *Riella notarisii* is known from only one location in the eastern part of the island [20] and it is one of the few Sicilian taxa of brackish water environments.

The mosses *Weissia levieri*, *Hydrogonium bolleanum,* and *Tortula freibergii* in Sicily grow in riparian environments or on the shore of rivers and streams such as the Belice River, in western Sicily, and the Fiumefreddo River, in the eastern Sicily.

In addition to the previously mentioned taxa, *Anthoceros agrestis* Paton, *Fissidens rivularis* (Spruce) Schimp., *Grimmia decipiens* (Schultz) Lindb., and *Philonotis rigida* Brid. are considered as Near Threatened in Italy and therefore require attention and population monitoring. Finally, *Sarmentypnum exannulatum* (Schimp.) Hedenäs should be the subject of specific investigations since there have been no reports in Sicily for 50 years or more [21].

**Figure 6:**
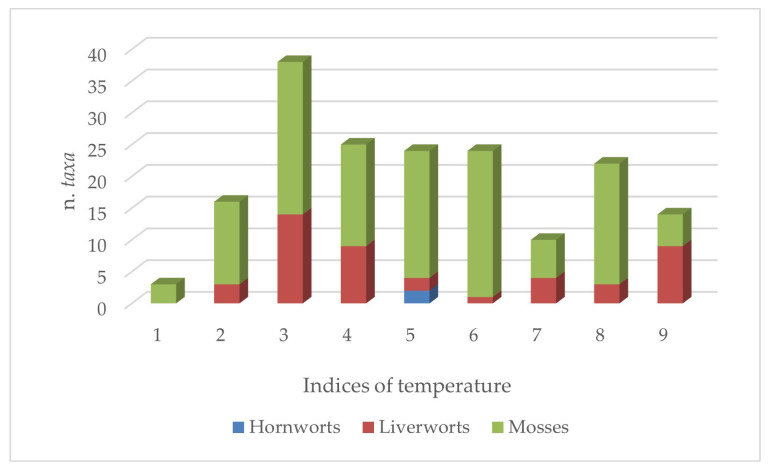
Consistency of group taxa of Sicilian aquatic and humid areas with different temperature indices.

**Figure 7:**
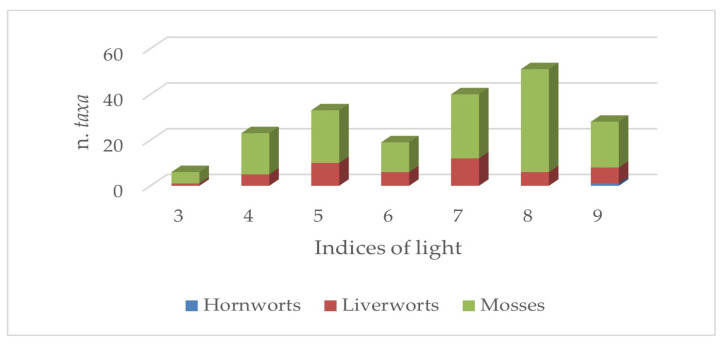
Consistency of group taxa of Sicilian aquatic and humid areas with different light indices.

**Figure 8:**
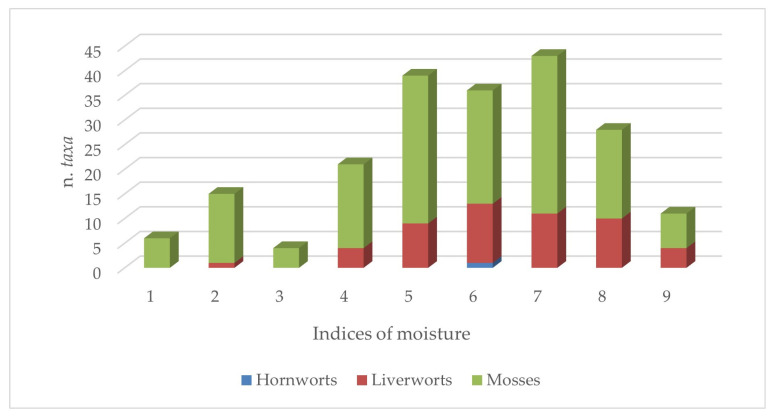
Consistency of group taxa of Sicilian aquatic and humid areas with different moisture indices.

The ecological patterns related to the factors that limit the growth of bryophytes, like temperature, light, and moisture, are shown in Figure 6, Figure 7 and Figure 8. Overall, it can be observed that the flora of the aquatic environments and humid areas of Sicily are quite diversified from an ecological point of view, as demonstrated by the patterns illustrated below.

With reference to temperature, the bryophytes under study are predominantly mesothermal (71 taxa overall with indices 4,5,6), but a marked microthermal component is also present (58 taxa), and the numbers of thermophilic and of indifferent taxa are also not negligible (42 and 32, respectively). This connotation is observed more for mosses than for liverworts and hornworts (Figure 6).

The analysis of the light factor pattern underlines the low presence of sciaphilous taxa in all the three subdivisions of bryophytes (only six taxa), and also a significant difference between mosses, on the one hand, and liverworts and hornworts, on the other hand (Figure 7). In particular for mosses, a high presence of heliophilous taxa is noted (93 *taxa*).

Regarding moisture (Figure 8), the majority of taxa are mesophilous species and then, obviously, hygrophilous and hydrophilous species (95 and 82 taxa overall).

**Figure 9:**
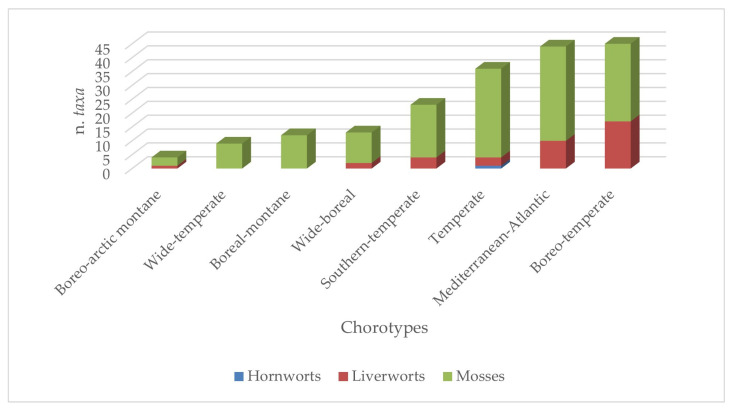
Chorological pattern of Sicilian bryoflora of the aquatic and humid habitats.

From a chorological point of view, as can be seen in Figure 9, an almost analogous occurrence of boreo-temperate taxa (45 taxa) and Mediterranean–Atlantic taxa (44 taxa) is observed, followed by the temperate component with 34 taxa. The three bryophyte groups contribute differently to this pattern. In fact, separately analyzing the pattern of liverworts and hornworts from the one of mosses, a rather clear prevalence of the boreo-temperate chorotype is noted in the first case, while mosses are almost equally represented by high numbers of Mediterranean–Atlantic, temperate, and boreo-temperate taxa. Lower numbers of taxa are recorded for the more typically boreal chorotypes, which overall contribute to a discrete chorological diversification of the flora.

**Figure 10:**
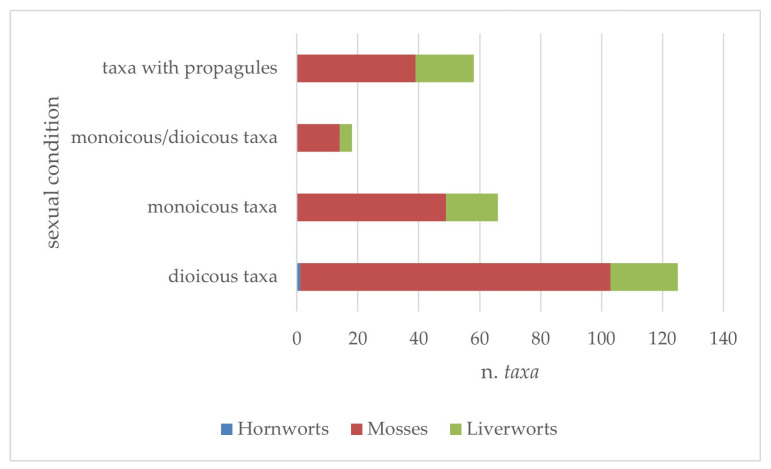
Reproductive biology data of Sicilian taxa of aquatic and humid habitats.

With reference to reproductive biology, from the data available in the literature, it emerged that the majority of taxa (124) are dioecious, with 102 species of mosses, 22 of liverworts, and 1 hornwort (Figure 10); monoecious species are also present with many taxa, of which 49 are mosses and 17 liverworts; finally, a not negligible number of taxa (58) include species that form propagules for vegetative reproduction. This pattern is also observed when analyzing the three bryophyte components separately.

## 3. Discussion

From the analysis of the results obtained during the survey, it is possible to gauge some general considerations on the component of the Sicilian bryophyte flora that lives in aquatic and humid areas. Evidently, these taxa are quite high in number and very diversified from a taxonomic point of view. This is a component that represents 36% of the Sicilian bryophyte flora and which remarkably contributes to determining its overall richness and diversity.

As regards the geographical distribution on the island, these taxa are mostly located in the riparian environments of watercourses, lakes, or ponds. However, in this respect, the following considerations should be made. A look at the literature on the different types of habitats suggests the need to increase research into some of them (e.g., gorges) that are often less accessible and therefore less explored than others, such as the main watercourses of the island [22,23,24]. On the shores of lakes and watercourses, affected by significant fluctuations in water level, now very accentuated in the different seasons, an increase in ubiquitous taxa, often also indifferent to ecological factors, is observed. In addition, the drastic decrease in precipitations to which Sicily has been subjected to in recent years would require frequent monitoring of many of these habitats, including the small peat bogs of the Madonie mountain range, where the only known locations of the genus *Sphagnum* in Sicily are found [25].

All rare taxa, especially the ones at risk of extinction, and taxa, although not very rare, that are specialists of aquatic and humid environments, such as the species of the genus *Fontinalis*, should at least be monitored in consideration of the ongoing climate changes that directly affect these habitats and therefore all the organisms that live there. Among these, in fact, bryophytes can particularly suffer the effects of habitat fragmentation and modification.

With reference to reproductive biology, fragility could concern the populations of both the numerous dioecious and monoecious taxa, which are well represented in these habitats. The former could be affected by global warming by encountering a lower fertilization rate due to a possible reduction in the number of individuals; the latter could have poor adaptability due to a lower genetic variety within the populations due to self-fertilization phenomena [26,27]. Furthermore, with reference to species that form asexual diasporas, while the production and germination processes are less sensitive to habitat quality and meteorological conditions than those for spore production [28,29], on the other hand, vegetative propagation reduces the ability to adapt to changing environmental conditions.

In the light of the above, monitoring of the presence of these taxa in Sicily may be useful in the future. Particular attention should be paid to rare taxa in Italy and, similarly, for many taxa, further studies aimed at improving, or in some cases acquiring, knowledge on the different aspects of their reproductive biology would be needed. More generally, the results obtained may be helpful in orienting future research, as well as in monitoring and protection actions for taxa and habitats.

Overall, there is an obviously emphasized need to extend the studies to the humid and aquatic sites of Sicily which are still little or not at all investigated from a bryofloristic point of view. This could create a picture of the bryophytes diversity of these fragile environments that is as complete as possible, which in the future will require more and more monitoring and protection measures.

## 4. Materials and Methods

This study analyses the bryophyte component of the aquatic and humid habitats of Sicily from a taxonomic, phytogeographic, biological, and ecological point of view. A list of the taxa known to date is provided, and their patterns are illustrated with reference to hornworts, liverworts and mosses. The list was extrapolated from the inventory of a total of 614 taxa (125 hornworts and liverworts and 489 mosses) known in Sicily, which is stored in the database of the Sicilian bryophyte flora, managed at the University of Palermo. In addition, the more recent scientific literature regarding the bryophytes of the environments under study was consulted, and the data reported for Sicily in the most updated check-list of Italian bryophytes [21] were taken into account as well. The check-list was also used as a reference for the nomenclature.

For the purposes of this investigation, after having identified the Sicilian aquatic and humid environments for which bryofloristic data were available, the different types of habitats were assembled into the following 5 groups: riparian habitats, peaty environments, drips and small waterfalls, gorges, and brackish water environments.

To conduct the ecological analyses, the factors that influence bryophyte growth the most were considered (temperature, light, and moisture). Ellenberg indicator values assigned to bryophytes by Düll [30] and recently updated by Hill et al. [31] were applied.

Chorological and biological traits (sexuality and presence of vegetative propagules) followed van Zuijlen [32], with the same abbreviations.

Furthermore, the recent red lists of Puglisi et al. [17,18] were considered to evaluate the conservation interest of the taxa, as well as the red list of European bryophytes [19], to which reference was made for all bryophytes. An analysis of the taxa distribution in Italy was carried out with reference to Aleffi et al. [21].

### Geographical, Bioclimatic, and Plant Landscape Features of Sicily

**Figure 11:**
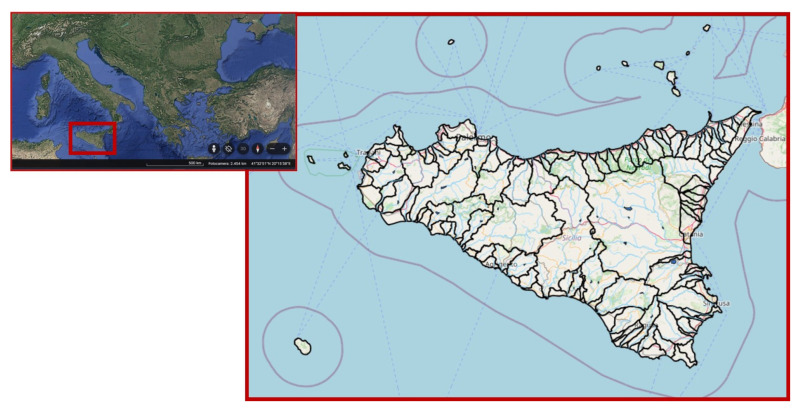
Sicilian area and hydrographic network of surface water. Modified by https://webgis.arpa.sicilia.it (accessed on 29 November 2024).

Located in the center of the Mediterranean basin, Sicily has a predominantly hilly and mountainous territory (Figure 11). The highest altitudes are reached in M. Etna (3340 m a.s.l.), in the eastern part of the island, and in the northern mountain ranges along the Tyrrhenian and Ionian coasts and are made up of the Madonie Mountains (with Pizzo Carbonara, 1979 m a.s.l.), Nebrodi Mountains (with M. Soro, 1847 m a.s.l.), and Peloritani Mountains (with Montagna Grande, 1374 m a.s.l.). Further south, in the western part, the isolated reliefs of Rocca Busambra (1615 m a.s.l.) and M. Cammarata (1579 m a.s.l.) stand out. The main forest formations of the island are located in all these reliefs and mountain systems [33]. Further representatives are those of deciduous woods of the *Quercus pubescens* group, and those of Holm oak woods, cork oak woods, and beech woods. Moreover, naturalistic and landscape importance have widely spread the communities in the territory represented by shrubs and garrigues. The south–central area, occupied by a sulfurous plateau, and the south-eastern area of the Iblei Mountains are instead the least wooded, as the most extensive cultivation systems are located here, mainly cereals and also pastures and trees.

As reported by Brullo and Spampinato [34], according to the classification of Rivas-Martínez [35], the territory is divided into the following bioclimatic belts: Inframediterranean (annual average temperature: 18–20 °C) along the southern slopes in the extreme south; Thermomediterranean (annual average temperature: 16–18 °C) along the coastal and sub-coastal belt of the various areas; Mesomediterranean (annual average temperature: 13–16 °C) in the hilly submountain areas; Supramediterranean (annual average temperature: 8–13 °C), on the highest peaks of the Sicani, the Madonie, the Nebrodi, the Peloritani Mts., and of Mt. Etna; Oromediterranean (annual average temperature: 4–8 °C) and Crioromediterranean (annual average temperature: 2–4 °C) in the summit of Mt. Etna.

From a hydrographic point of view, Sicily hosts different types of aquatic and humid habitats, both lentic (coastal wetlands, temporary ponds, lakes, reservoirs) and lotic (springs, streams, permanent and seasonal watercourses), often with many rare species [36]. Although rather modest in flow and extension, the Sicilian rivers form a quite complex network which extends across the entire region. The network is made up of watercourses which mostly have a torrential flow and a very variable flow rate throughout the year, as a consequence of the orography and general climatic conditions of the island. The high vegetation covering the riparian and aquatic habitats is well known. The shores and the areas that are periodically flooded are covered by ripisilvae vegetation, with discontinuous communities characterized by willows (*Salix alba* L., *S. purpurea* L. s.l., *S. pedicellata* Desf. e *S. gussonei,* Brullo and Spamp.), poplars (*Populus nigra* L. and *P. alba* L.), and, in eastern Sicily, also by *Platanus orientalis* L., which in the quarries of the Iblei area gives rise to forest formations unique in Italy, comprising *Salici pedicellatae Platanetum orientalis,* Barbagallo et al. (1979). In rivers and, in general, in the riverbeds subject to frequent drying out, scrub vegetation is more frequently found, including *Nerium oleander* L. and *Tamarix* sp. pl. More specifically, aquatic communities are instead constituted above all by herbaceous species of the genera *Ranunculus* L., *Mentha* L., and *Polygonum* L., and also by rushes, sedges, and reeds.

The main rivers of the island in terms of flow and extension are today protected in reserves or natural parks, such as the recently established Alcantara River Park.

Natural lentic environments are today very reduced in number, extension, and flow [37] as a consequence of the various actions of exploitation of the aquifers, reclamation and alteration of the hydrological regime of the tributaries, and also due to the increasingly prolonged periods of drought that are recorded on the island. Among them it is worth mentioning the so-called “margi”, small and today very limited marsh environments, located in the Madonie Mountains area, which are peculiar from the bryofloristic point of view due to the presence of the genus *Sphagnum*, absent in the rest of the island.

In addition to the few natural environments that still survive, there are artificial habitats that in some cases play similar ecological roles by hosting species of interest, as in the case reported by Campisi et al. [15] for the moss species *Physcomitrium patens*.

## Figures and Tables

**Table 1:** Numerical consistency of bryophyte taxa of Sicilian aquatic and humid habitats.

	Mosses	Liverworts	Hornworts	Taxa tot.
n. specific and infraspecific taxa	167	55	2	224
n. families	28	26	1	55
n. genera	75	29	2	106

## Data Availability

The original contributions presented in the study are included in the article/Supplementary Material, further inquiries can be directed to the corresponding author.

## Supplementary Materials

The following supporting information can be downloaded at: https://www.mdpi.com/article/10.3390/plants14020199/s1, File S1: Lists of bryophytes taxa of aquatic and humid habitats of Sicily with relatives biological, ecological and chorological features.
